# Tethered Alkylammonium Dications as Electrochemical Interface Modifiers: Chain Length Effect on CO_2_ Reduction Selectivity at Industry-Relevant Current Density

**DOI:** 10.1021/acsami.4c04632

**Published:** 2024-05-29

**Authors:** Walter A. Parada, Urban Sajevic, Rashad Mammadzada, Pavlo Nikolaienko, Karl J. J. Mayrhofer

**Affiliations:** † HI ERN (IEK-11), Forschungszentrum Jülich GmbH, Erlangen 91058, Germany; ‡ Department of Chemical and Biological Engineering, Friedrich-Alexander-Universität Erlangen-Nurnberg (FAU), Erlangen 91054, Germany

**Keywords:** CO_2_, electroreduction, copper, electrocatalysis, modifiers, real time, GDE

## Abstract

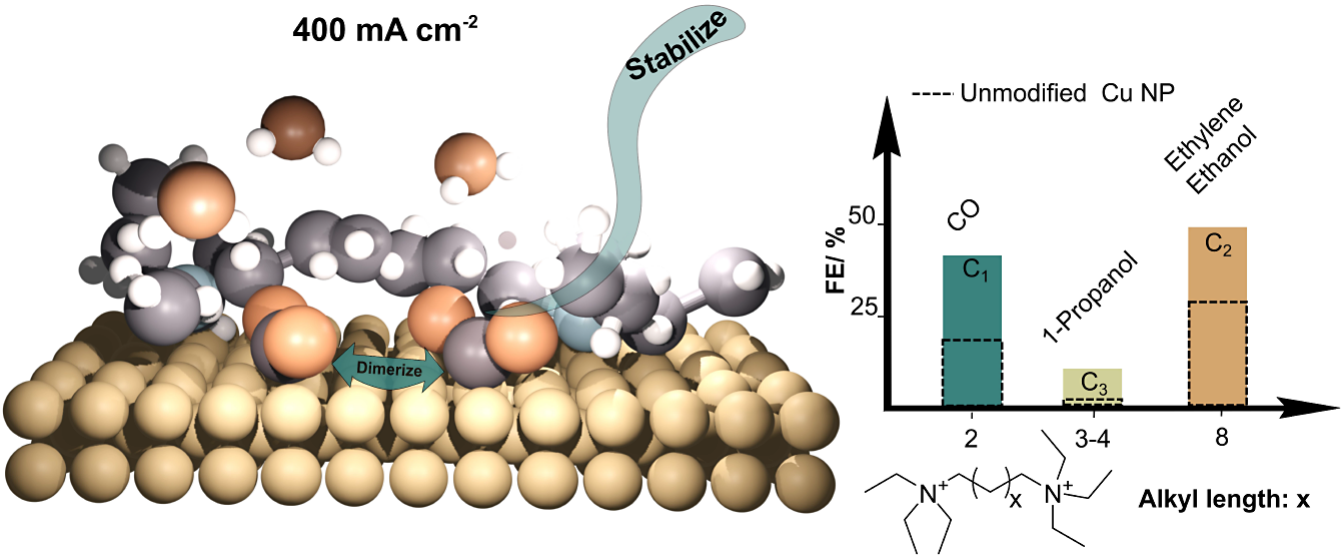

The electrochemical reduction of CO_2_ (CO_2_RR) has the potential to be an economically viable method to produce platform chemicals synergistically with renewable energy sources. Copper is one of the most commonly used electrocatalysts for this purpose, as it allows C–C bond formation, yielding a broad product distribution. Controlling selectivity is a stepping stone on the way to its industrial application. The kinetics of the reaction can be modified to favor the rates of certain products quickly and inexpensively by applying additives such as ionic liquids and coelectrolytes that directly affect the electrocatalytic interface. In this work, we propose tethered tetraalkylammonium salts as double-charged cationic modifiers of the electrochemical double layer to control CO_2_RR product selectivity. A novel setup comprising a gas diffusion electrode (GDE) flow cell coupled with real-time mass spectroscopy was used to study the effect of a library of the selected salts. We emphasize how the length of an alkyl linker effectively controls the selectivity of the reaction toward C_1_, C_2_, or C_3_ products at high relevant current densities (*J*_total_ = −400 mA cm^–2^) along with the inhibition of the parasitic hydrogen evolution reaction. Standard long-term experiments were performed for quantitative validation and stability evaluation. These results have broad implications for further tailoring an effective catalytic system for selective CO_2_ reduction reaction.

## Introduction

The electrochemical reduction of carbon dioxide (CO_2_RR) is a compelling process that combines the intermittent nature of renewable electricity and the sustainable production of chemical feedstock and fuels.^1,2^ Over the last few decades, numerous important achievements have been made to embrace a carbon-neutral circular economy.^3,4^ In order to compete against fossil-fuel-based hydrocarbon cracking, CO_2_ electrolyzers are expected to have a selected product partial current density of several hundred mA cm^–2^. To achieve such a milestone, electrolyzers with gas diffusion electrodes (GDEs) or membrane electrode assemblies (MEAs) are commonly employed, circumventing the mass transport limitation.^5^ Nevertheless, the future of industrial CO_2_RR will be determined by developing readily available and effective catalytic systems that fit a broad set of requirements, which are extremely dependent on the catalyst of choice.

The reaction selectivity is often prioritized due to the additional costs involved in separation. For this reason and due to the prolonged stability of bismuth and silver catalysts, the technology readiness level (TRL) of the electrochemical conversion of CO_2_ to single-carbon chemicals like formic acid or carbon monoxide is rather advanced.^6,7^ Copper (Cu) is one of the few catalysts that inherently enables the generation of C–C bondings that lead to highly valued multicarbon products, such as ethanol, 1-propanol, and ethylene, depending on the applied potential.^8−11^ However, Cu electrodes are often related to fast deactivation and a broad liquid and gaseous product distribution. A number of Cu-based catalysts with varying compositions and morphologies have been investigated extensively to improve the product selectivity and catalyst lifespan, ranging from the nanoparticles with a specific ratio of crystal facets to the sputtered fibers with a high surface area and porosity (Table S1).^12,13^ Despite evident improvements, the catalyst stability is still too weak to be applied on an industrial scale. In addition to macroscopic deactivation involving carbonation and the flooding of GDEs, the copper lattice has shown evidence of self-reorganization, possibly due to the adsorbate-induced ballbot-type movement.^14^

Beyond the identity and morphology of the catalyst, the interface structure between the electrode surface and a local electrolyte composition or pH has a direct impact on CO_2_RR performance. According to Koper et al., no products are detected on gold in the absence of alkali cations.^15−17^ Increasing the cation size of the electrolyte can boost CO_2_RR and inhibit the hydrogen evolution reaction (HER), shifting the selectivity toward CO and C_2_ products, as evidenced by the number of reports in the literature.^15,16,18^ While the precise mechanism of this phenomenon is not yet fully understood, several explanations have been proposed. Of these proposals, the cation-involved coordinative stabilization of CO_2_RR-adsorbed intermediates has recently garnered interest.^19,20^ Altering the composition of the double layer and electrode environment opens up the possibility of tailoring the performance of the catalytic system. The latter was used as a pivotal point to build up the present study.

As the number of alkali cations is restricted, and the solubility of multivalent inorganic cation carbonates is low, an alternative application of water-soluble, positively charged organic molecules under CO_2_RR conditions has received considerable attention.^21−24^ For instance, tetraalkylammonium salts alter the electrode–electrolyte interface due to their weak coordinating ability, favoring the selectivity shift toward CO_2_RR, which so far is noticeable for CO on silver catalysts and formate on copper.^25^ As in the case of surfactants, changing the length of the alkyl chains could modulate hydrophobicity, carbon/carbon coupling, and other effects, altering the availability of interfacial water.^26−29^

This manuscript bridges the need of selectivity control and stability in Cu-based catalysts with the essential role of cations during CO2RR. Here, we report on the effect of symmetrical monoammonium and tethered diammonium bromides as coelectrolytes on copper-catalyzed CO_2_ electrolysis on GDEs for the first time. In this context, we propose that the tethering of weakly coordinating tetraalkylammonium dications provides an extra degree of freedom to modify an electrochemical interface as they interact with the C–C bonds formed on the Cu surface. Some examples of the usage of such salts could be traced to a Monsanto process of acrylonitrile electrodimerization and enhancing the stability of the lead electrode during organic electrosynthesis applications,^30−32^ which—to the best of our knowledge—have not previously been studied under CO_2_RR. Unlike prior studies where monocharged ammonium cations often hindered C_2+_ product formation on Cu, our findings demonstrate a positive effect, as evidenced by the data in Table S2. We employed both conventional chromatography and real-time analysis techniques with subsecond time resolution to monitor the broad product distribution. Additionally, impedance spectroscopy provided deeper mechanistic insights into the process.

## Results and Discussion

### Effect of Diammonium Salts on the Selectivity of the Cu Catalyst

The choice of the alkyl group at the ammonium centers for the design of dications was inspired by previous reports for the mechanistically similar CO reduction reaction (CORR). Li et al.^33^ made the qualitative observation in a differential electrochemical mass spectrometry (DEMS) setup that tetraalkylammonium bromides influence the CORR product distribution by varying the length of all four substituents simultaneously. The methyl and ethyl substituents displayed the greatest affinity toward ethylene production during CORR, while chain elongation was found to be detrimental for reduction until being fully blocked using tetraoctylammonium bromide. The latter study is also relevant for CO_2_RR, as both reduction reactions share similar mechanism that leads to the formation of carbon bonding.

**Figure 1:**
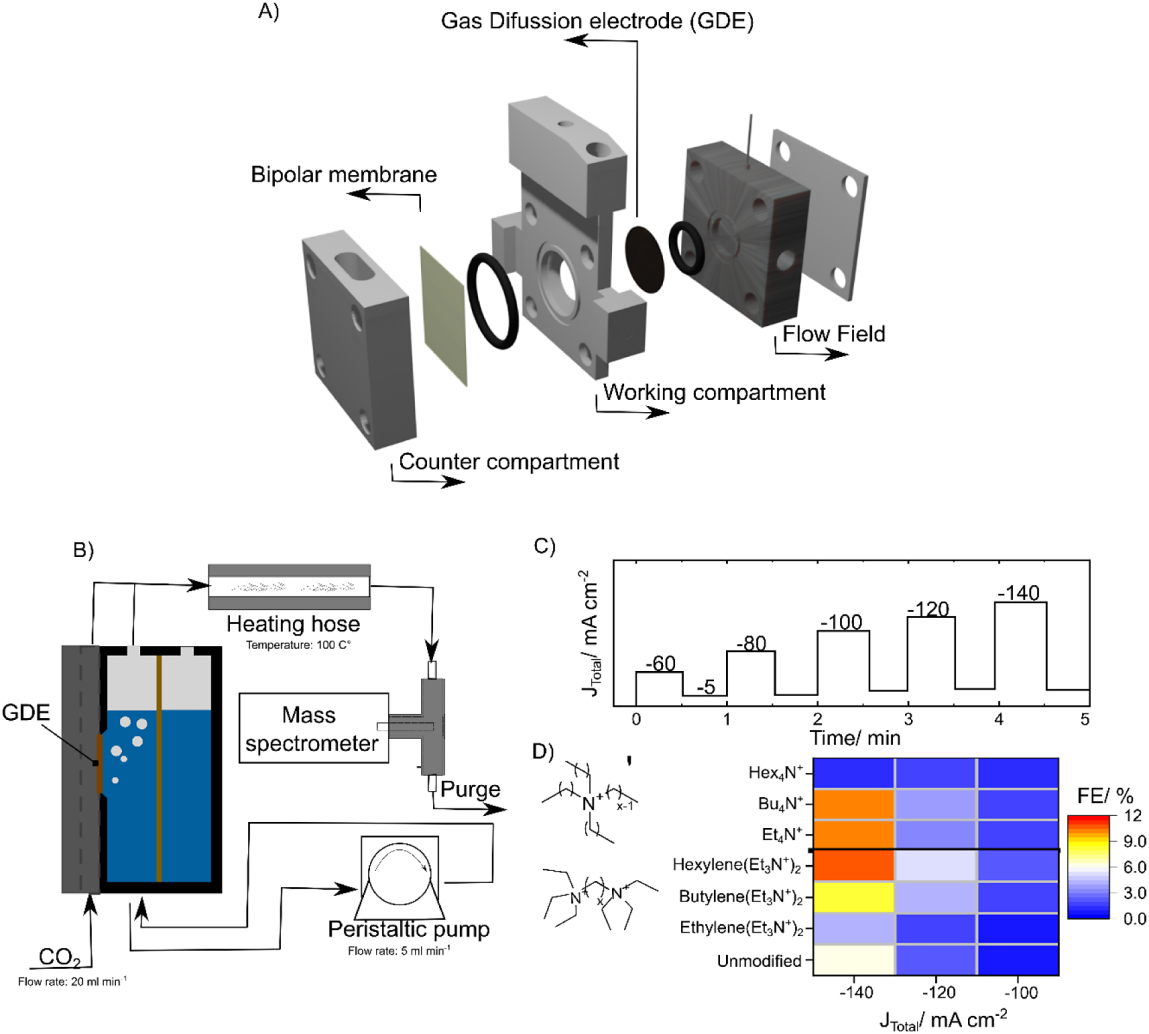
(A) Detailed configuration of the in-house designed GDE cell used for the electrochemical experiments (B) Schematic representation of the GDE setup coupled with online product analysis used for the scanning experiments.(C) Electrochemical protocol applied to generate heat maps of the (D) FE toward ethylene using tetraalkyl ammonium and diammonium salts. The chemical formula of the cations used is positioned alongside its corresponding plots. Unmodified is designated to the experiments on Cu without additives. Electrode: 1.3 mg cm^–2^ of Cu nanoparticles (20–30 nm). Electrolyte: 1 M KHCO_3_. Additive: 10 mM.

The effect of ammonium cations on CO_2_RR on copper nanoparticles was evaluated according to the selectivity toward ethylene using a GDE setup (Figure 1A). For quick operation, the electrochemical cell was coupled with a proton transfer reaction (PTR) mass spectrometer calibrated for ethylene detection (Figure S1), which allowed for product analysis in real time (Figure 1B). Aqueous solutions of various ammonium bromide salts with a concentration of 10 mM in 1 M KHCO_3_ were used to compare their effect. To highlight the influence of the additives as a function of their alkyl chain compared to an unmodified catalyst, it was sufficient to use a constant current protocol consisting of five different steps (−60 mA cm^–2^, – 80 mA cm^–2^, – 100 mA cm^–2^, – 120 mA cm^–2^, – 140 mA cm^–2^) for 30 s (Figure 1C). However, it was necessary to maintain the negative (−5 mA cm^–2^) current between the pulses to avoid the oxidation of the electrode. Copper has remarkable stability at negative potentials, as it minimizes the chances of restructuring.^34^ However, scanning electron microscope images of fresh and used catalysts evidenced a softening of the surface roughness after the electrolysis (Figure S8).

Similarly to Li’s report, we tested their influence on the reduction of CO_2_ in a GDE setup, ultimately leading to the same qualitative conclusion (Figure 1D). The highest faradaic efficiency (FE) was observed for tetrabutyl and tetraethylammonium bromides, while tetraethylammonium inhibited the formation of ethylene completely. Ethyl functionality for the diammonium salts was therefore selected due to its better synthetic practicability and lower molecular weight compared to the butyl.A set of diammonium bromide salts, whose structure was varied across the distance between the charged species, was synthesized from readily available precursors. Diammonium salts with a linker longer than octylene were found to be impractical due to foam formation during electrolysis, restricting the electrode area and flooding the gas outlet tubing.

The heat map of Figure 1D displays the faradaic efficiency calculated from the MS signal response for the diammonium salts. The FE toward ethylene increased as the chain size of the connecting alkyl unit became longer, and the current turned more negative. Ethylene production is negligible at current densities less negative than −100 mA cm^–2^. The highest FE in this set of experiments was recorded at −140 mA cm^–2^, with a maximum of 11% for hexyl, and the lowest at 5.6% and 5.4% for the unmodified catalyst and ethyl, respectively. The kinetics of faradaic processes follow an exponential relation with the overpotential as in the Butler–Volmer equation. Considering CO_2_RR has more favorable kinetics (steeper Tafel slope) than hydrogen evolution (HER), there should be a tendency toward ethylene formation as the potential becomes more negative. On polycrystalline copper, ethylene formation thus reaches a peak at approximately −1.05 V v*s.* RHE, which is mainly caused by mass transport limitations.^10,35^ However, other products such as methane start forming at this potential range, which could also influence the selectivity trends and the eventual decline of ethylene at more negative potentials. The trend in Figure 1D supports the idea that the ethylene FE would increase even further at higher current densities.

To study the influence of the additives on HER, a common parasitic side reaction, the gaseous product stream was also analyzed by an electron impact ionization mass spectrometer operated in real time (Figure S2). A concentrated solution of the diammonium salt **6** (octylene linker) was injected during a constant current step at −200 mA cm^–2^, with the resulting additive concentration equal to 10 mM. The fast effect response from the modifier presence, which was observed by a sudden increase in ethylene FE after the injection (Figure S3), indicates perturbations in their double layer rather than the creation of a passivation layer.

### Long-Term Electrolysis

**Figure 2:**
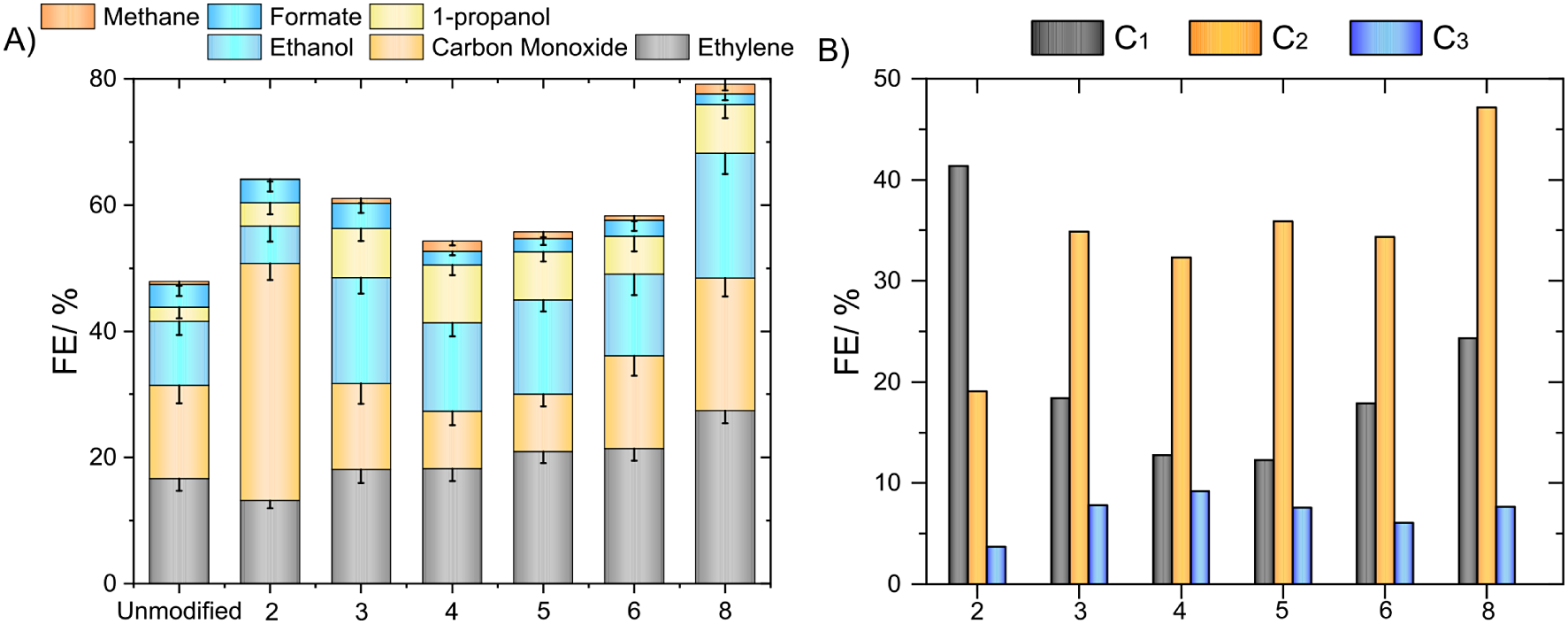
(A) FE of CO_2_RR reaction products individually and (B) organized according to the total number of carbon atoms. The digits in *x*-axis represent number of CH_2_ units between two ammonium headgroups in the corresponding dications. Unmodified is designated to the experiments on Cu without additives. Note that the sum of the faradaic efficiencies only displays the contribution of the CO_2_RR. The remaining efficiency is destined to the evolution of hydrogen (Figure S4). Unmodified is designated to the experiments on Cu without additives. Current density: – 400 mA cm^–2^. Electrolyte: 1 M KHCO_3_. Additive: 10 mM.

Confirmed upon brief screening, the enhancement of CO_2_RR selectivity toward ethylene in the presence of the diammonium salts was then tested further at an industry-relevant current density of −400 mA cm^–2^ for extended operation times. The design and electrolyte composition of the GDE cell remain the same as those used for screening tests. To close charge balance, it was important to quantify all products during the experiments. To this end, the gaseous outlet stream was analyzed by an inline GC, while the liquid products were sampled and quantified after the experiments by NMR. The FEs of the individual products and their sets, categorized by carbon number, are displayed in Figure 2A,B.

Starting with the C_1_ products, their formation rates are boosted for all modifiers, compared to the unmodified copper, and they change in an inverse parabolic trend within the alkyl chain length of the linker. The lowest rate is observed for the pentylene-linked modifiers and the highest rate for the ethylene-linked modifiers. This can be explained by the difference in the ability to stabilize the initial *CO_2_^–^ intermediate for the CO_2_RR. Methane contributes less than 5% of the FE, which could be attributed to its high overpotentials on Cu. Nevertheless, it exhibits the greatest increase in terms of percentage compared to the unmodified catalysts. The highest production of CO among all the experiments corresponds to a 41% FE with the ethylene modifier, doubling the value from the blank catalyst. At the same time, formate displayed the fastest production using short alkyl chains (ethylene and propylene). Its production is the least affected by the presence of modifiers, exhibiting FEs smaller than or equal to 7%. Both CO and formate require the same number of electron transfers with the highest selectivity at potentials lower than −0.7 V vs. E_RHE_ on polycrystalline copper, which is relatively small compared to ethylene and methane.^10^ Initially, it was assumed they share the same *COOH intermediate.^36,37^ However, according to later studies, their mechanisms are separated, attributing the generation of the formate to *OOCH^38,39^ or the reaction of *H with CO_2_.^40^ This explains the discrepancies in formate and CO or methane formation upon using the diammonium cations in this study.

The FEs of the C_2_ products, as screened in the preliminary tests for ethylene, exhibited an almost linear increase with the chain length of the connecting alkyl unit. For instance, the ethylene FE went from 14% to 28% for dications with ethylene and octylene linkers, respectively. Further elongation to dodecyl did not affect its selectivity at 10 mM loading. With respect to ethanol, the most significant change is noticeable for the modifiers with ethylene and propylene linkers, where the selectivity increased approximately three times the highest FE for ethanol was 20% compared to 11% for the unmodified catalysts. Looking closely, the rates at which ethanol and ethylene production is affected by diammonium modifiers substantially differ, which suggests that they do not share the same reaction pathway under the given conditions. However, there is no contradiction as the CO_2_RR mechanism bifurcates, as supported by experimental evidence and theoretical calculations.^11,41,42^ For instance, carbene-like intermediates *CH_2_ are able to circumvent the *CO dimerization process that is usually attributed as an intermediate to oxygenated products.^43^ Overall, the selectivity toward C_2_ products is enhanced by all modifiers, except for the ethylene-linked CO-selective one.

Under the given reaction conditions, 1-propanol (PrOH) was detected as the only C_3_ product. Compared to the FE from the bare Cu catalyst (<1%), all modifiers increased PrOH production to more than 5%, with the peak achieved for butylene linker (10% FE). Considering the high currents applied, it is a remarkably large value, comparable only with a few specialized catalysts.^44−46^ These findings are again consistent with the hypothesis that the conformational flexibility of diammonium dications plays a significant role in stabilizing and providing the required physical space for CH_2_CHCHO*, a key intermediate for C_3_ products.^47^ Nevertheless, more studies are needed to navigate through possible multiple schemes for double-layer perturbations.

As the octylene linker displayed the greatest selectivity to CO_2_RR, analogous *N*-methylpiperidin-based, *N*-methylpyrollidin-based, and quinoclidine-based diammonium dications were also tested. Quaternary nitrogen aliphatic cycles are more rigid than trimethylammonium units, which were expected to act differently. However, no major improvement in the product distribution was observed compared to the blank catalysts (Figure S7), highlighting the importance of the steric bulk volume of the ammonium headgroup.

The effect of the loading of diammonium cations on CO_2_RR was studied at −400 mA cm^–2^ (Figure S5) and a constant ionic strength of the electrolyte of 0.5. As observed in the long experiments, 10 mM of the organic additive (roughly 1% molar) is sufficient to detect the influence, as is consistent with reported surface coverage under OCP for monocationic surfactants.^28^ Nevertheless, a further replacement of the supporting electrolyte with diammonium cations was tested. At the end of the set experiments, K^+^ was extremely diluted. The selectivity to ethylene was improved differentially, reaching a maximum for the pure diammonium salt as the only supporting electrolyte. This enhancement, however, comes with a drastically decreased electrolyte ohmic conductivity, compromising its industrial applicability.

**Figure 3:**
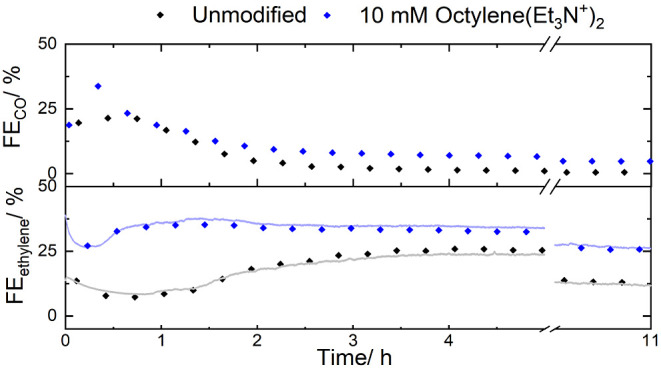
Faradaic efficiency for carbon monoxide and ethylene production for modified (blue) and unmodified (black) Cu NP catalyst at GDE (20–30 nm, 1.3 mg cm^–2^) over 10-h operation. The continuous line represents the mass spectrometer product detection; 1 M KHCO_3_, 10 mM octylene additive; *J* = −400 mA cm^–2^.

Besides the overall activity, the stability of the modified catalyst is also an important parameter. We therefore conducted a CO_2_RR experiment with 10 mM octylene dication in catholyte for a 10-h operation. While an inline GC (Figure 3) was used to monitor gas product formation for the entire duration of the experiment, the real-time MS was employed to track transient changes over the first 30 min. It is worth emphasizing that the use of Cu catalyst is intrinsically limited by its poor long-term stability, which originates from electrode flooding. For this reason, the present study highlights the differences that the organic additives cause to a standard unoptimized Cu catalyst. While these differences are significant, they are, unfortunately, still far from industrial standards.

The first couple of minutes reveal the most changes in selectivity, which can be seen using MS detection (Figure S6). The reaction starts with a high FE toward ethylene, which decreases steadily for 2 and 60 min for the modified and unmodified catalysts, respectively. This transient behavior might be due to the change in GDE macroproperties, such as wettability or interfacial viscosity, including the effect of the formation of alcohols. However, further experimentation is required to understand the dynamics at the reaction interface. After this point, the efficiency reaches a maximum and slowly decays. In the case of the modified catalysts, the activation is faster, while the decay is gradual. A substantial increase in ethylene formation (13%) also accompanies the enhanced stability in the presence of tested diammonium cations. The drift in the potential with the modifier during the experiment remained almost constant, increasing by just 0.1 V. We attribute the enhanced stability to the long hydrophobic alkyl chains that inhibit carbonation, as the Helmholtz outer plane has a high concentration of ammonium cations. The size of the cations makes the precipitation of carbonates less favorable than for potassium.

### Mechanistic Considerations

Although the unified mechanism for the cation effect is the subject of intensive discussion, it is clear that near-surface cation organization directly affects the double-layer properties. In contrast to well-defined, spherical hydrated alkali cations, the structuring effect of nonsymmetric tetraalkylammonium salts on double layer formation is yet to be addressed.

Thoi et al. therefore studied the effect of trimethyl(alkyl)ammonium surfactants on copper by electrochemical impedance spectroscopy (EIS) and IR.^28^ They observed an increase in charge transfer resistance and a decrease in the double-layer capacitance (C_DL_) across the alkyl chain length. This was attributed to a double-layer disorganization and the low dielectric permittivity of the alkyl chains. Noteworthy, the C_DL_ concentration dependence exhibited a local minimum, which can be explained by the gradual reordering of the double-layer structure within the increased presence of ammonium cations, as revealed by Stark probe near-field IR spectroscopy. However, opposite trends were reported for Ag electrode material results, where charge transfer resistance was substantially lower in the presence of CO_2_ than for HER. By taking into account outstanding CO formation selectivity for Ag compared to Cu, it is clear that the electrochemical interface structure is determined not only by electrolyte–electrode interaction but that it also involves CO_2_RR-adsorbed intermediates.

The alkylene-tethered diammonium bromides are particularly interesting in such a context, as they carry two spatially separated positive charges per molecule. Electrochemical impedance spectroscopy (EIS) was used (see Supporting Information for details) for copper to gain further insights. We emphasize that polycrystalline copper was chosen to average the influence of the organic additives on multiple Cu crystal structures. In this way, we could neglect the surface restructure that copper might undergo during the reaction. Moreover, energy dispersive X-ray analysis on the composition of the catalysts after the reaction displayed that the catalysts remained as Cu, and no nitrogen intrusion into the lattice was observed (Figure S8).

**Figure 4:**
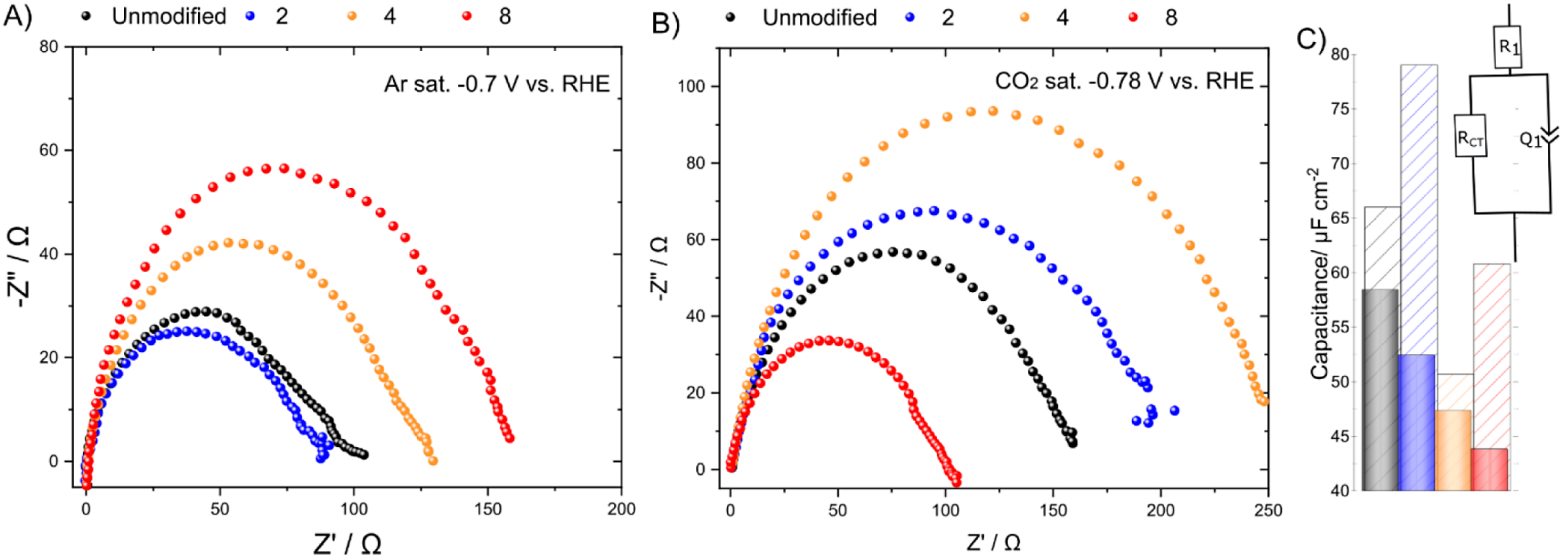
Nyquist plot of EIS experiments conducted in (A) argon and (B) CO_2_-saturated electrolyte using diammonium salts in an H cell. The digits in top-label represent number of CH_2_ units between two ammonium headgroups in the corresponding dications. (C) Capacitance fitted to the given equivalent circuit from the experiments from A (dashed lines) and B (filled columns) in 1 M KHCO_3_ and 10 mM of the additive. Unmodified is designated to the experiments on Cu without additives.

In a first set of experiments, the electrolyte was saturated with argon to assess the effect of the organic modifiers on HER without the presence of CO_2_. At potentials from −0.4 to −0.8 V vs. E_RHE_, the charge transfer resistance (*R*_CT_) increases monotonically within the length of the alkyl linker (Figure 4), similar to previous reports.^24^ Surface-populated weakly coordinating ammonium units displace alkali cations from the double layer, disrupting interfacial water mass transfer and causing HER suppression. The ethylene-linked modifier appears to be exceptional, most likely due to the increased charge density of the molecule.

In the case of the CO_2_-saturated buffer (Figure 4B), the relations of the observed changes in *R*_CT_ across varied chain lengths are more complex with respect to both HER and CO_2_RR. In such a case, the corresponding charge transfer resistance is a function of the number of electron transfers required to form the variety of adsorbed intermediate species. While *R*_CT_ steadily increases until the maximum for the butylene linker, further chain elongation results see the *R*_CT_ decline to values lower than those of bare copper for linkers longer than hexylene.

The double-layer capacitance change of all diammonium salts, in contrast to that reported for monoammonium cations, tended to be nonsystematic within the chain length variation under HER conditions (Figure 4C). At the same time, under CO_2_RR conditions, the double-layer capacitance of all diammonium salts displays an apparent, almost linear decay as a function of the applied electrode potential and the chain length from 58 F cm^–2^ of blank copper to 43 F cm^–2^ of octylene diammonium bromide, respectively. Unlike the reported behavior of surfactants on copper, the corresponding dications express an increased disorder in the double layer, which could be attributed to the alkyl chain’s degree of freedom to the system.

These observations indicate a strong dipolar interaction between adsorbed diammonium and CO_2_-derived intermediates. The latter directly supports an enhanced total activity toward CO_2_RR upon dynamic electrode modification. However, the changes in the rate-determining step, surface charge density, and interface water acidity cannot be ruled out.

**Figure 5:**
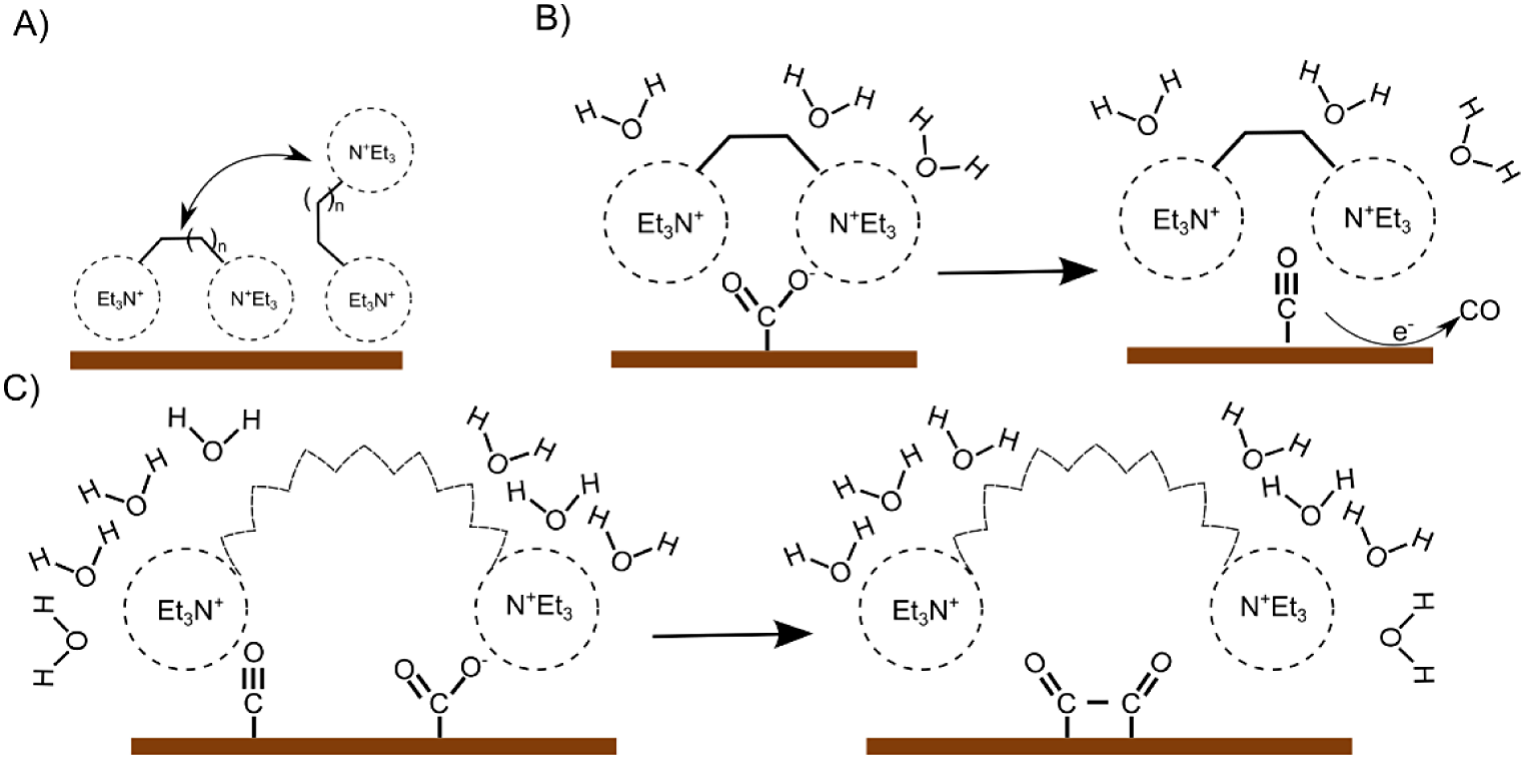
(A)Proposed mechanism with which the diammonium salts stabilize CO_2_ intermediates for alkyl substituents with (B) small and (C) large chain lengths.

Considering the ability of alkylammonium cations to be part of the inner Helmholtz plane (IHP), we hypothesized two modes of their surface orientation analogous to the dynamically formed assembly of ammonium-based surfactants on Ag catalysts during CO_2_RR. The order of the surfactant, where the positive heads pointed toward the negatively charged electrodes, increased as the potential became more negative. The first “perpendicular” mode involves only one ammonium facing toward the electrode surface, with the other charged side oriented to the electrolyte. Excess anions and the noncovalent interactions between hydrophobic linker chains can stabilize such a configuration. The second mode, however, indicates that both charged headgroups are participating in IHP, resulting in “horizontal” coordination, which is thermodynamically more favorable due to the increased entropy factor (Figure 5A). Previous experimental results using monocharged quaternary ammonium salts exhibit purely vertical orientation, which resulted in predominant formate production. The absence of formate in our experiments gives further proof to the proposed horizontal orientation (Table S2). The increasing hydrophobicity across the alkyl chain might enhance the aggregation of diammonium salts in the double layer. However, their actual surface orientation also depends on the presence of surface CO_2_RR intermediates due to their electrostatic interactions with the ammonium headgroup. The highest CO selectivity of the ethylene-linked diammonium cation can thus be attributed to a horizontal orientation, which stabilizes the corresponding CO_2_* intermediate and restricts dimerization due to its steric bulk (Figure 5B). Further elongation of a linker brings more conformational allowance, resulting in mixed modes of surface orientation that affect interfacial water and CO_2_ mass transfers (Figure 5B). The observed selectivity trends are in agreement with the EIS data, supporting the orientation change during CO_2_RR. Further elaboration of such an intriguing hypothesis is currently ongoing. Molecular dynamics simulation could give a better overview of the possible arrangements

## Conclusions

A set of linked bis-ammonium dibromides as electrode microenvironment modifiers were assessed for CO_2_RR. We tested the influence of the size of their alkyl substituents to define the distance between the two nitrogen atoms. The study reveals that these organic modifiers enhance CO_2_RR over hydrogen evolution reaction, resulting in an  above 75% for ethylene and octylene linkers on standard copper nanoparticles at industrially relevant current densities (up to −400 mA cm^–2^). We attribute the selectivity increase to the quaternary nitrogen dications stabilizing key CO_2_ intermediates and the controlled repulsion of water molecules to the hydrophobic alkyl chains on the Cu surface. Adding to current state of the role of cations during CO_2_RR, we could experimentally see the process happening with only the presence of the ammonium salts in absence of alkali cations.

Interestingly, the CO_2_RR selectivity toward C_1_, C_2_, and C_3_ products is controlled immediately upon adding organic modifiers, indicating changes in the double-layer structure. C_2_ products tend to form as the length of the modifier becomes larger (FE_C2+_ ≈ 40% for the octylene diammonium linker), while C_1_ products display the opposite trend (FE_C1_ ≈ 40% for the ethylene diammonium linker). C_3_ product formation shows a local maximum for organic modifiers possessing alkyl chains with similar molecular sizes (propyl to butyl), reaching an FE of 10%. Trends in CO_2_RR product distribution, supported by EIS data, suggest that the diammonium surface orientation depends on the linker chain length and the adsorbed CO_2_RR intermediates. Moreover, ethylene production under high current densities reaches a stable value faster and decays much slower in electrolytes modified with the diammonium salts than in untreated catalysts.

The results presented herein demonstrate that the electrocatalytic performance of reactions can be finely tuned by employing low concentrations of diammonium salts. It is highly recommended to explore this concept within ionomers or by directly bonding to the Cu catalyst surface in alternative cell designs devoid of electrolytes (MEA). Further investigations are required to understand the mechanism of double-layer perturbations at the atomic level for a predictive structure–activity relationship (SAR) evaluation, which is a topic for the follow-up research.

## Experimental Part

### Preparation of the Catalysts

For the catalysts, Cu NPs (100 mg) with a size of 20–30 nm (Sigma) were mixed with isopropanol (3.18 mL) and Nafion (20%w Sigma) (25 mg) to form an ink, which was dispersed using an ultrasonication horn (Branson 150) for 20 min and 40% of intensity. The latter was sprayed (TAMIYA HG Airbrush III 0.3 mm) onto a 5.5 × 3 cm carbon paper (Freudenberg 23B) on a surface heated surface (70C). The final catalyst loading was equal to 1.3 mg CuNP cm^–2^ with 4% Nafion.

### General Procedure for Synthesizing Bromide Anions Containing Diammonium Cations and Alkyl Linkage Chains of Length “N″

Reagents and solvents were used as received from the supplier. The corresponding 1,n dibromoalkane (58 mmol) was reacted with triethylamine (46.5 g, 465 mmol) in acetonitrile (30 mL) and stirred for 24 h at 60 °C under an inert atmosphere (argon). The organic solvent and the excess of trimethylamine were removed in vacuo using a rotary evaporator. The resulting solid material was then dissolved in ethanol (15 mL) and recrystallized using ethyl acetate. The crystals were dried overnight under a high vacuum at 60 °C to obtain a white powder.

**2**. 1,2-bis(triethylammonium)ethane dibromide. ^1^H NMR (chloroform-d6, 600 MHz):δ1.4 (t, 18 H), 3.44–3.5(q, 12 H), 3.61–3.66 (m, 4 H).

**3**. 1,3-bis(triethylammonium)propane dibromide 1H NMR (chloroform-d6, 600 MHz):δ1.4 (t, 18 H),1.72–1.78 (q, 2 H), 3.54–3.55(m, 12 H), 3.86–3.91(m, 4 H)

**4**. 1,4-bis(triethylammonium)butane dibromide 1H NMR (chloroform-d6, 600 MHz):δ1.39 (t, 18 H),1.70–1.71 (m, 4 H), 3.45–3.55 (m, 12H) 3.5–3.67 (m, 2 H).

**5**. 1,5-bis(triethylammonium)pentane dibromide 1H NMR (chloroform-d6, 600 MHz):δ1.38 (t, 18 H),1.66–1.73 (q, 2 H), 2.05–2.12(q, 4 H), 3.4–3.51 (m, 12H) 3.57–3.63 (m, 2 H).

**6**. 1,6-bis(triethylammonium)hexane dibromide 1H NMR (chloroform-d6, 600 MHz):δ1.37 (t, 18 H),1.57–1.66 (m, 4 H) 1.94–2.03(m, 4 H), 3.44–3.57 (m, 16 H).

**8**. 1,8-bis(triethylammonium)octane dibromide 1H NMR (chloroform-d6, 600 MHz):δ1.37 (t, 18 H),1.46–1.54 (m, 8 H) 1.81–1.90(m, 4 H), 3.56–3.46 (m, 16 H).

**12**. 1,12-bis(triethylammonium)dodecane dibromide 1H NMR (deuterium oxide-d6, 600 MHz):δ1.19 (t, 18 H),1.33–1.22 (m, 16 H) 1.56–1.65(m, 4 H), 3.05–3.11 (m, 4 H), 3.17–3.24 (q, 12 H).

### Electrochemical Measurements

Electrochemical measurements were conducted in an in-house-developed two-compartment and three-electrode configuration cell compatible with a gas diffusion electrode. The cell material was PTFE for the electrolyte containers and graphite for the flow field. The electrolytes in the two half-cells were separated by a bipolar membrane (Fuel Cell Store). The latter provided an appropriate mechanical stability and ensured a stable pH gradient across the cell during the operation. The electrochemical measurements were controlled by a potentiostat (SP 240, BioLogic), an Hg/HgO reference electrode (BASi), an Ir-MMO counter electrode(Metakem), and the respective working electrode with an exposed area of 1 cm^2^.

Steady-state electrolysis was performed in 1 M KHCO3 and 10 mM of the organic modifiers unless specified. The 15 mL electrolyte volume of the working compartment was recirculated with a flow rate of 20 mL min-1 using a peristaltic pump (ISMATEC, REGLOICC) to avoid the possible accumulation of gas bubbles or liquid products at the electrode’s surface. The flow field was fed with a constant CO_2_ rate of 20 mL min–1. All electrolytes were prepared in ultrapure water (18.2 MOhm cm, a totalorganic carbon level below 4 ppb; Milli-Q IQ 7000, Merck), and the gas flow was controlled by a mass flow controller (EL-FLOW Prestige, Bronkhorst). Simultaneously, a mass flow meter measured the outlet gas stream (CORI-FLOW, Bronkhorst). The latter ensured a precise product flow estimation regardless of the ever-changing properties of the gas mixture. All measurements were conducted under ambient conditions. The electrolysis was performed (if not specified) for 1 h and repeated three times to ensure reproducibility.

### Product Analysis

The outlet of the working electrode half-cell was connected to an online GC (Clarus 580, Arnel Engineered, PerkinElmer). The gas phase products were quantified by a thermal conductivity detector and a flame ionization detector during steady-state electrolysis. The gas separation was performed on a hayesep N (NR021501, PerkinElmer) and a molecular sieve 13X (NR022501, PerkinElmer) with Ar as a carrier gas with a flow rate of 30 mL min^–1^. During electrolysis, the GC gas injections were performed at 2, 20, 38, and 56 min, and the calculation of the faradaic efficiency used the average value of all injections and the outlet flow rate.

After 1 h of current holds, the catholyte was collected to quantify the liquid products. For this purpose, 500 mL of the electrolyte with the electrolysis products was mixed with a 500 mL solution of 10 mM dimethyl sulfoxide (DMSO) in deuterated water after the hour-long electrolysis. The mixture was analyzed using ^1^D^1^H NMR (600 MHz, JEOL). Standard curves were made using ethanol, formate, and 1-propanol over the concentration range of interest. The internal standards used DMSO in 1 M KHCO3, and the water peak was suppressed by a presaturation sequence. All NMR parameters used were identical for the quantification and calibration procedures. The peak area ratios from DMSO and the products allowed product quantification compared to standard curves.

The partial current density, *j*_*i*_, and the faradaic efficiency, FE_*i*_, for each product are calculated in the following way:

For gases
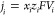
1

For liquids
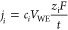
2
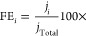
3where *x_i_* represents the volume fraction of the products measured via the online GC using an independent calibration standard gas; *z*_*i*_ is the number of electrons needed to obtain a given product, *V*_m_ represents the molar CO_2_ gas flow rate that was evaluated at the moment of the injection, *c_i_* is the molar concentration detected in the NMR, *V*_WE_ is the volume of the working electrode, *t* is the total electrolysis time, and *F* is the Faraday constant.

### Real-Time Mass Spectroscopy

The gaseous products were split into two separate gas streams. One led to the online gas chromatography set; the other to one of the mass spectrometers.

The proton-transfer-reaction time-of-flight mass spectrometer, coupled with the selective reagent ionization technology (PTR/SRI-TOF-MS Ionicon), has a PEEK capillary inlet of 1/16 heated by a heating hose at 80 *C*°. The sample flow rate is determined by the internal pressure of the instrument, which operates with multiple vacuum pumps and without any valves for the sample path. The quantification (Supporting Information) mainly focuses on ethylene recording the signal of primary water ions and ethylene at 22 *m*/*z*.

EI-QMS A 1/16’ capillary led the gas sample to an ionization chamber, which was ionized by a robust Ir filament (Pfeiffer) and subsequently evaluated in a quadrupole detector equipped with an electrode multiplier (Pfeifer HiCUBE). The sample flow rate is regulated by a complex interplay of the pressure of a preconditioning chamber and the main ionization chamber. At 20 mL min^–1^ of CO_2_ for the main experiments, the pressure remained at 10^–5^ and 10^–7^ dPa, respectively. The quantifications of the gaseous stream were only achieved by comparing the raw spectrometer output with the already calibrated GC response.

## Supporting Information Available

Additional information on the mass spectroscopy methods, the faradaic efficiencies of hydrogen evolution, the performance of other functional groups for the organic dications in CO_2_RR, and real-time product quantification (PDF).

## References

[ref1] Dinh C.-T.; Burdyny T.; Kibria M. G.; Seifitokaldani A.; Gabardo C. M.; García de Arquer F. P.; Kiani A.; Edwards J. P.; De Luna P.; Bushuyev O. S.; Zou C. CO_2_ electroreduction to ethylene via hydroxide-mediated copper catalysis at an abrupt interface. Science 2018, 360 (6390), 783–787. 10.1126/science.aas9100. 29773749

[ref2] de Vasconcelos B. R.; Lavoie J.-M. Recent advances in power-to-X technology for the production of fuels and chemicals. Front. Chem. 2019, 7, 392 10.3389/fchem.2019.00392. 31231632 PMC6560054

[ref3] Maginn E. J. What to Do with CO2. ACS Publ. 2010, 1, 3478–3479. 10.1021/jz101582c.

[ref4] Tan X.; Yu C.; Ren Y.; Cui S.; Li W.; Qiu J. Recent advances in innovative strategies for the CO _2_ electroreduction reaction. Energy Environ. Sci. 2021, 14 (2), 765–780. 10.1039/D0EE02981E.

[ref5] Sánchez O. G.; Birdja Y. Y.; Bulut M.; Vaes J.; Breugelmans T.; Pant D. Recent advances in industrial CO_2_ electroreduction. Curr. Opin. Green Sustain. Chem. 2019, 16, 47–56. 10.1016/j.cogsc.2019.01.005.

[ref6] Iqbal M. Z.; Imteyaz S.; Ghanty C.; Sarkar S. A review on electrochemical conversion of CO_2_ to CO: Ag-based electrocatalyst and cell configuration for industrial application. J. Ind. Eng. Chem. 2022, 113, 15–31. 10.1016/j.jiec.2022.05.041.

[ref7] Ewis D.; Arsalan M.; Khaled M.; Pant D.; Ba-Abbad M. M.; Amhamed A.; El-Naas M. H. Electrochemical reduction of CO2 into formate/formic acid: A review of cell design and operation. Sep. Purif. Technol. 2023, 316, 123811 10.1016/j.seppur.2023.123811.

[ref8] Hori Y.; Takahashi R.; Yoshinami Y.; Murata A. Electrochemical reduction of CO at a copper electrode. J. Phys. Chem. B 1997, 101 (36), 7075–7081. 10.1021/jp970284i.

[ref9] Takahashi I.; Koga O.; Hoshi N.; Hori Y. Electrochemical reduction of CO2 at copper single crystal Cu (S)-[n (111)×(111)] and Cu (S)-[n (110)×(100)] electrodes. J. Electroanal. Chem. 2002, 533 (1–2), 135–143. 10.1016/S0022-0728(02)01081-1.

[ref10] Kuhl K. P.; Cave E. R.; Abram D. N.; Jaramillo T. F. New insights into the electrochemical reduction of carbon dioxide on metallic copper surfaces. Energy Environ. Sci. 2012, 5 (5), 7050–7059. 10.1039/c2ee21234j.

[ref11] Schouten K. J. P.; Qin Z.; Koper M. T. Two pathways for the formation of ethylene in CO reduction on single-crystal copper electrodes. J. Am. Chem. Soc. 2012, 134 (24), 9864–9867. 10.1021/ja302668n. 22670713

[ref12] Tabassum H.; Yang X.; Zou R.; Wu G. Surface engineering of Cu catalysts for electrochemical reduction of CO2 to value-added multi-carbon products. Chem. Catl. 2022, 2, 1561–1593. 10.1016/j.checat.2022.04.012.

[ref13] Nitopi S.; Bertheussen E.; Scott S. B.; Liu X.; Engstfeld A. K.; Horch S.; Seger B.; Stephens I. E.; Chan K.; Hahn C.; No̷rskov J. K. Progress and perspectives of electrochemical CO2 reduction on copper in aqueous electrolyte. Chem. Rev. 2019, 119 (12), 7610–7672. 10.1021/acs.chemrev.8b00705. 31117420

[ref14] Vavra J.; Ramona G. P.; Dattila F.; Kormányos A.; Priamushko T.; Albertini P. P.; Loiudice A.; Cherevko S.; Lopéz N.; Buonsanti R. Solution-based Cu+ transient species mediate the reconstruction of copper electrocatalysts for CO_2_ reduction. Nat. Catal. 2024, 7, 89–97. 10.1038/s41929-023-01070-8.

[ref15] Monteiro M. C.; Dattila F.; López N. R.; Koper M. T. The role of cation acidity on the competition between hydrogen evolution and CO2 reduction on gold electrodes. J. Am. Chem. Soc. 2022, 144 (4), 1589–1602. 10.1021/jacs.1c10171. 34962791 PMC8815072

[ref16] Gu J.; Liu S.; Ni W.; Ren W.; Haussener S.; Hu X. Modulating electric field distribution by alkali cations for CO2 electroreduction in strongly acidic medium. Nat. Catal. 2022, 5 (4), 268–276. 10.1038/s41929-022-00761-y.

[ref17] Monteiro M. C.; Dattila F.; Hagedoorn B.; García-Muelas R.; López N.; Koper M. T. Absence of CO_2_ electroreduction on copper, gold and silver electrodes without metal cations in solution. Nat. Catal. 2021, 4 (8), 654–662. 10.1038/s41929-021-00655-5.

[ref18] Ayemoba O.; Cuesta A. Spectroscopic evidence of size-dependent buffering of interfacial pH by cation hydrolysis during CO2 electroreduction. ACS Appl. Mater. Interfaces 2017, 9 (33), 27377–27382. 10.1021/acsami.7b07351. 28796478

[ref19] Malkani A. S.; Anibal J.; Xu B. Cation effect on interfacial CO2 concentration in the electrochemical CO_2_ reduction reaction. ACS Catal. 2020, 10 (24), 14871–14876. 10.1021/acscatal.0c03553.

[ref20] Shin S.-J.; Choi H.; Ringe S.; Won D. H.; Oh H.-S.; Kim D. H.; Lee T.; Nam D.-H.; Kim H.; Choi C. H. A unifying mechanism for cation effect modulating C1 and C2 productions from CO2 electroreduction. Nat. Commun. 2022, 13 (1), 5482 10.1038/s41467-022-33199-8. 36123326 PMC9485141

[ref21] Tao Z.; Wu Z.; Wu Y.; Wang H. Activating copper for electrocatalytic CO2 reduction to formate via molecular interactions. ACS Catal. 2020, 10 (16), 9271–9275. 10.1021/acscatal.0c02237.

[ref22] Zhong Y.; Xu Y.; Ma J.; Wang C.; Sheng S.; Cheng C.; Li M.; Han L.; Zhou L.; Cai Z.; Kuang Y. An artificial electrode/electrolyte interface for CO2 electroreduction by cation surfactant self-assembly. Angew. Chem. 2020, 132 (43), 19257–19263. 10.1002/ange.202005522. 32686265

[ref23] Zhang Z.-Q.; Banerjee S.; Thoi V. S.; Shoji Hall A. Reorganization of interfacial water by an amphiphilic cationic surfactant promotes CO_2_ reduction. J. Phys. Chem. Lett. 2020, 11 (14), 5457–5463. 10.1021/acs.jpclett.0c01334. 32524821

[ref24] Ge W.; Chen Y.; Fan Y.; Zhu Y.; Liu H.; Song L.; Liu Z.; Lian C.; Jiang H.; Li C. Dynamically formed surfactant assembly at the electrified electrode–electrolyte interface boosting CO_2_ electroreduction. J. Am. Chem. Soc. 2022, 144 (14), 6613–6622. 10.1021/jacs.2c02486. 35380035

[ref25] Berto T. C.; Zhang L.; Hamers R. J.; Berry J. F. Electrolyte dependence of CO2 electroreduction: Tetraalkylammonium ions are not electrocatalysts. ACS Catal. 2015, 5 (2), 703–707. 10.1021/cs501641z.

[ref26] Banerjee S.; Zhang Z.-Q.; Hall A. S.; Thoi V. S. Surfactant perturbation of cation interactions at the electrode–electrolyte interface in carbon dioxide reduction. ACS Catal. 2020, 10 (17), 9907–9914. 10.1021/acscatal.0c02387.

[ref27] Mohandas N.; Narayanan T. N.; Cuesta A. Tailoring the Interfacial Water Structure by Electrolyte Engineering for Selective Electrocatalytic Reduction of Carbon Dioxide. ACS Catal. 2023, 13, 8384–8393. 10.1021/acscatal.3c01223.

[ref28] Banerjee S.; Han X.; Thoi V. S. Modulating the electrode–electrolyte interface with cationic surfactants in carbon dioxide reduction. ACS Catal. 2019, 9 (6), 5631–5637. 10.1021/acscatal.9b00449.

[ref29] Kong K.; Li A.-Z.; Wang Y.; Shi Q.; Li J.; Ji K.; Duan H. Electrochemical carbon–carbon coupling with enhanced activity and racemate stereoselectivity by microenvironment regulation. Nat. Commun. 2023, 14 (1), 6925 10.1038/s41467-023-42724-2. 37903827 PMC10616095

[ref30] Edinger C.; Waldvogel S. R. Electrochemical deoxygenation of aromatic amides and sulfoxides. Eur. J. Org. Chem. 2014, 2014 (24), 5144–5148. 10.1002/ejoc.201402714.

[ref31] Edinger C.; Grimaudo V.; Broekmann P.; Waldvogel S. R. Stabilizing lead cathodes with diammonium salt additives in the deoxygenation of aromatic amides. ChemElectrochem 2014, 1 (6), 1018–1022. 10.1002/celc.201402050.

[ref32] Kulisch J.; Nieger M.; Stecker F.; Fischer A.; Waldvogel S. R. Efficient and stereodivergent electrochemical synthesis of optically pure menthylamines. Angew. Chem., Int. Ed. 2011, 50 (24), 5564–5567. 10.1002/anie.201101330. 21567696

[ref33] Li J.; Li X.; Gunathunge C. M.; Waegele M. M. Hydrogen bonding steers the product selectivity of electrocatalytic CO reduction. Proc. Natl. Acad. Sci. U. S. A. 2019, 116 (19), 9220–9229. 10.1073/pnas.1900761116. 31004052 PMC6511002

[ref34] Raaijman S. J.; Arulmozhi N.; Koper M. T. Morphological stability of copper surfaces under reducing conditions. ACS Appl. Mater. Interfaces 2021, 13 (41), 48730–48744. 10.1021/acsami.1c13989. 34612038 PMC8532114

[ref35] Hori Y.; Murata A.; Takahashi R. Formation of hydrocarbons in the electrochemical reduction of carbon dioxide at a copper electrode in aqueous solution. J. Chem. Soc., Faraday Trans. 1989, 85 (8), 2309–2326. 10.1039/f19898502309.

[ref36] Peterson A. A.; Abild-Pedersen F.; Studt F.; Rossmeisl J.; No̷rskov J. K. How copper catalyzes the electroreduction of carbon dioxide into hydrocarbon fuels. Energy Environ. Sci. 2010, 3 (9), 1311–1315. 10.1039/c0ee00071j.

[ref37] Calle-Vallejo F.; Koper M. T. Theoretical considerations on the electroreduction of CO to C2 species on Cu (100) electrodes. Angew. Chem. 2013, 125 (28), 7423–7426. 10.1002/ange.201301470. 23733719

[ref38] Todorova T. K.; Schreiber M. W.; Fontecave M. Mechanistic understanding of CO_2_ reduction reaction (CO2RR) toward multicarbon products by heterogeneous copper-based catalysts. ACS Catal. 2020, 10 (3), 1754–1768. 10.1021/acscatal.9b04746.

[ref39] Chernyshova I. V.; Somasundaran P.; Ponnurangam S. On the origin of the elusive first intermediate of CO_2_ electroreduction. Proc. Natl. Acad. Sci. U. S. A. 2018, 115 (40), E9261–E9270 10.1073/pnas.1802256115. 30224482 PMC6176638

[ref40] Cheng T.; Xiao H.; Goddard Iii W. A. Reaction mechanisms for the electrochemical reduction of CO_2_ to CO and formate on the Cu (100) surface at 298 K from quantum mechanics free energy calculations with explicit water. J. Am. Chem. Soc. 2016, 138 (42), 13802–13805. 10.1021/jacs.6b08534. 27726392

[ref41] Santatiwongchai J.; Faungnawakij K.; Hirunsit P. Comprehensive mechanism of CO_2_ electroreduction toward ethylene and ethanol: The solvent effect from explicit water–Cu (100) interface models. ACS Catal. 2021, 11 (15), 9688–9701. 10.1021/acscatal.1c01486.

[ref42] Chen P.; Wu Y.; Rufford T. E.; Wang L.; Wang G.; Wang Z. Organic molecules involved in Cu-based electrocatalysts for selective CO_2_ reduction to C2+ products. Mater. Today Chem. 2023, 27, 101328 10.1016/j.mtchem.2022.101328.

[ref43] Chang B.; Pang H.; Raziq F.; Wang S.; Huang K.-W.; Ye J.; Zhang H. Electrochemical reduction of carbon dioxide to multicarbon (C 2+) products: Challenges and perspectives. Energy Environ. Sci. 2023, 16 (11), 4714–4758. 10.1039/D3EE00964E.

[ref44] Kim D.; Kley C. S.; Li Y.; Yang P. Copper nanoparticle ensembles for selective electroreduction of CO_2_ to C2–C3 products. Proc. Natl. Acad. Sci. U. S. A. 2017, 114 (40), 10560–10565. 10.1073/pnas.1711493114. 28923930 PMC5635920

[ref45] Wang H.; Matios E.; Wang C.; Luo J.; Lu X.; Hu X.; Li W. Rapid and scalable synthesis of cuprous halide-derived copper nano-architectures for selective electrochemical reduction of carbon dioxide. Nano Lett. 2019, 19 (6), 3925–3932. 10.1021/acs.nanolett.9b01197. 31034237

[ref46] Peng C.; Luo G.; Zhang J.; Chen M.; Wang Z.; Sham T.-K.; Zhang L.; Li Y.; Zheng G. Double sulfur vacancies by lithium tuning enhance CO2 electroreduction to n-propanol. Nat. Commun. 2021, 12 (1), 1580 10.1038/s41467-021-21901-1. 33707465 PMC7952561

[ref47] Pablo-García S.; Veenstra F. L.; Ting L. R. L.; García-Muelas R.; Dattila F.; Martín A. J.; Yeo B. S.; Pérez-Ramírez J.; López N. Mechanistic routes toward C 3 products in copper-catalysed CO _2_ electroreduction. Catal. Sci. Technol. 2022, 12 (2), 409–417. 10.1039/D1CY01423D.

