# Supplementary material for: Tethered Alkylammonium Dications as Electrochemical Interface Modifiers: Chain Length Effect on CO2 Reduction Selectivity at Industry-Relevant Current Density
Source: ACS Appl Mater Interfaces. 2024 May 29;16(23):30107–16. doi: 10.1021/acsami.4c04632 (PMC11181265; doi:10.1021/acsami.4c04632)
Supplement: Supplementary file 1 — am4c04632_si_001.pdf [file am4c04632_si_001.pdf]

Supporting information

Tethered Alkylammonium Dications as Electrochemical Interface Modifiers: Chain Length Effect on CO_2 Reduction Selectivity at Industry-Relevant Current Density

Walter Agustin Parada, Urban Sajevic , Rashad Mammadzada, Pavlo Nikolaienko*, Karl J. J. Mayrhofer**

Forschungszentrum Jülich GmbH, HI ERN (IEK-11), Germany

***Department of Chemical and Biological Engineering, Friedrich-Alexander-Universität Erlangen-Nurnberg (FAU), Germany*

Corresponding Author E-mail: p.nikolaienko@fz-juelich.de

TABLE S1 Performance of various Cu-based catalyst for the electroreduction of CO_2. The choice of studies was driven by keeping Cu as the base-material itselft, so oxides and metal alloys or mixtures were excluded.

Cu-based catalyst	FE/ % C_2H_4	CO	Alcohols	Potential/ V vs. RHE	Current density/ mA cm^{-2}	Source
Polycristaline	26	1	6	-1	5	1
Cu (100)	32	0	10	-1	5	1
Cu(110)	14	0	7	-1	5	1
Cu (111)	5	0	1	-1	5	1
Cu dopped with borum	52	0	27	-1	70	2
Cu dopped with Hydrogen	22	10	10	-1	51	2
Cu dopped with Carbon	33	8	16	-1	70	2
Cu nanocubes (44 nm)	44	2	25	-1.1	6	3
Cu dopped with Fluoride/GDE	57	10	20	-0.7	800	4
Cu dopped with Iodide/GDE	30	15	10	-0.7	370	4
Cu/GDE	30	15	10	-0.7	200	4
Plasma treated nanocubes	45	0	22	-1	35	5
Cu3N	66	0	12	-1	57	6
Catalyst ionomer planar heterojunction (CIPH)/GDE	60	9	-	-	850	7

TABLE S2 Comparison of the performance of Cu catalyst modified with quaternary-ammonium salts present in the electrolyte for the electrochemical reduction of CO_2: Study Results vs. Literature

Ammonium modifier on Cu	FE/ % C_2H_4	CO	Alcohols	Potential/ V vs. RHE	Current density/ mA cm^{-2}	Source
Tetrame-thylammoniumchloride	38	20	9	-0.8	>10	8
Cetyltrimethylammoniumchloride	25	18	19	-0.8	>10	8
Cetyltrimethylammoniumbromide	0	0	0	-0.5	2	9
Cetyltrimethylammoniumbromide	5	12	0	-1.05	>10	10
Cetyltrimethylammoniumchloride	0	7	0	-0.8	6	11
**Bis(triethylammonium)ethylene dibromide**	**13**	**38**	**10**	**<-0.8**	**400**	**This work**
**Bis(triethylammonium)octane dibromide**	**27**	**19**	**38**	**<-0.8**	**400**	**This work**

Calibration PTR-GDE setup

The high-resolution mass spectrometer proton transfer reaction (PTR) utilizes H_3O^+ molecules as an ionization source. It can efficiently ionize various compounds, particularly volatile organic compounds (VOCs), as long as their proton affinity is higher than that of water. For instance, the ethylene is ionized according to the following reaction:

$$H_3O^+ + C_2H_4 \rightarrow C_2H_2^+ + H_2O$$

The product quantification in concentration units (C) is achieved by multiplying the ratio of the primary (H_3O^+) and the targeted compound's "i" ions multiplied and constants related to the sample acquisition and other kinetic parameters according to the equation:

$$C_i[mol\ cm^{-3}] = \frac{1}{\eta \cdot k \cdot t} \frac{S_i}{S_{water}}$$

Here, t and k are the drift tube's reaction time and the ionization reaction rate, respectively. η is the collection efficiency, which represents how much of the products from the stream are actually being measured.

A calibration gas provided by Air liquid with 5 %m of ethylene and pure CO_2 was mixed using Bronkhorst mass flow meters(El-flow) to produce different ethylene concentrations. The concentration mixtures ranged from 1 to 1100 ppm of Ethylene, keeping the total flow rate constant at 20 ml min^{-1}. The PTR signal response of the various mixtures is displayed in Figure S1.

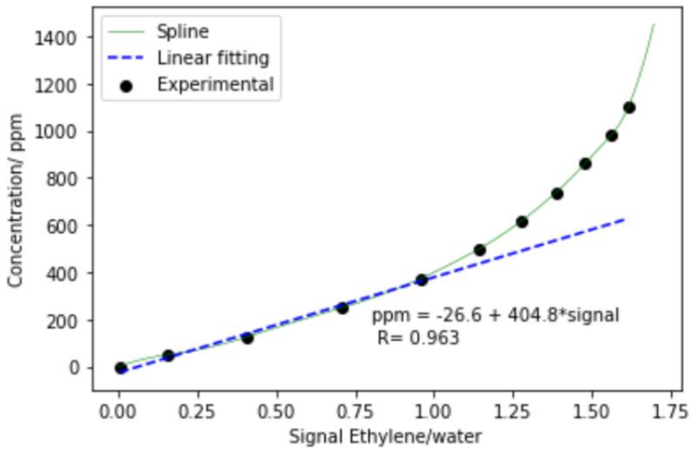

Figure S1. Ethylene calibration curve for multiple ethylene concentrations using the PTR GDE setup. The dotted line fits the ethylene-water signal ratio within a linear region, and the green curve fits all points by cubic splines. The ratio corresponds to the ratio of the ethylene $C_2H_2^+$ (m/z=26) and H_3O^+ (m/z=21) signal of mass. H_3O^+ with a mass of 21 was used, corresponding to only 0.206% of the total isopical abundance of water molecules.

Fitting the experimental points in Figure S1 with a line enabled the quantification of ethylene at any given time, allowing real-time measurements. However, using the non-linear range was avoided to not deplete the primary ions and prevent instrument damage.

The partial current density of ethylene was calculated as follows:

$$(2)\ i_{ethylene} = C \cdot \overline{V} \cdot n \cdot F$$

Where n is the number of electrons needed to convert CO_2 to ethylene, V is the flow rate, and F is the Faraday constant. The quantification of organic volatile compounds was not considered as they were not the focus of the study.

Calibration EI-GC-GDE set-up

Unlike the high-resolution PTR-MS, an electron impact mass spectrometer can ionize hydrogen and methane. To obtain quantitative results, the mass spectrometer and the gas chromatography unit were run simultaneously, as shown in Figure S1. The flame ionization detector's signal responses determined the hydrogen concentration and the ethylene and methane thermal conductivity detector. Each experiment was calibrated, forming a calibration curve based on the GC quantification. For example, a long-term experiment with 15 GC injections. As shown in the figure, plotting the signal responses of both instruments together allows the line fitting, enabling real-time product quantification. The concentration $C_{GC}$is proportional to the normalized mass spectrometer signal $(S - S_{background})$.

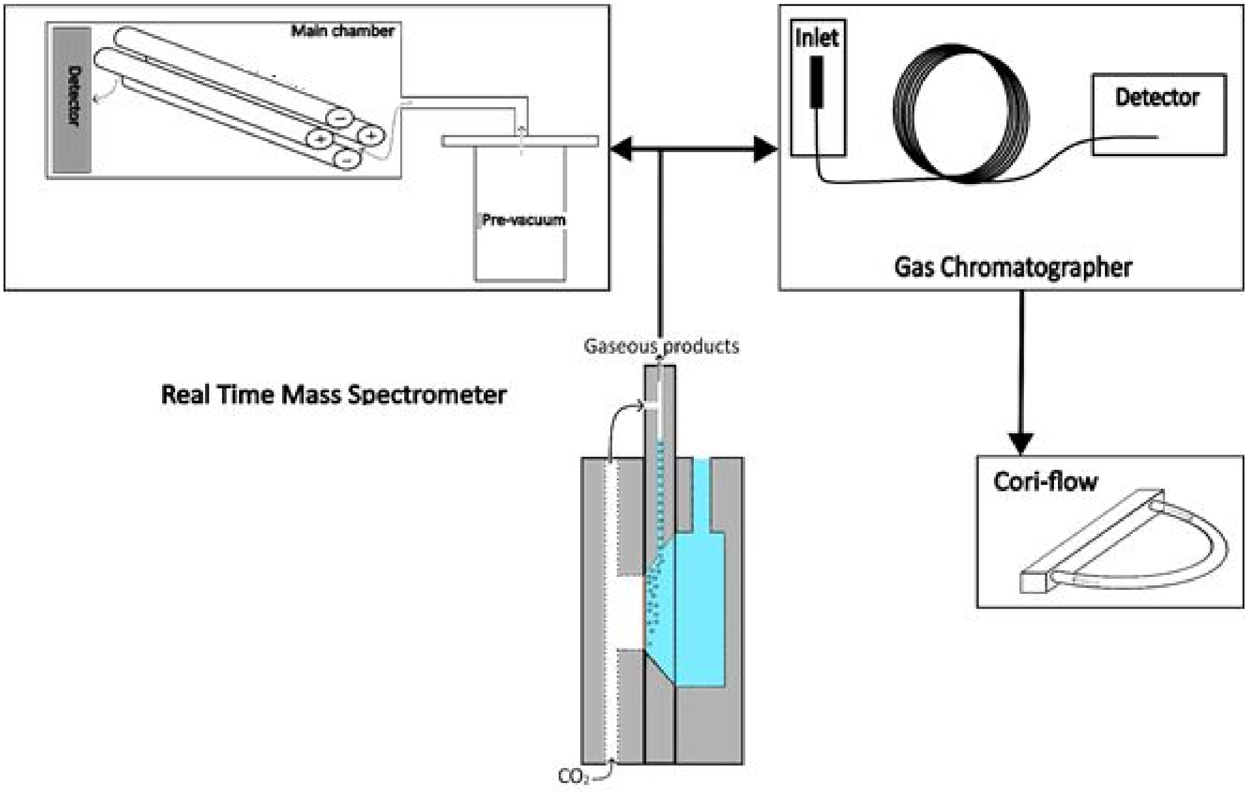

Figure S2. A simplified diagram of the EI-GC-GDE setup during a CO_2RR experiment shows the arrows denoting the gas products' flow from the GDE cell outlet to the electrode impact mass spectrometer and the gas chromatography.

Utilizing a mass flow meter based on the Coriolis effect (Cori-Flow), allowed a more accurate estimation of the outstream cell flows. It consists of a vibrating tube through which the fluid flows. As the fluid moves through the tube, it causes the tube to twist or deform due to the Coriolis force

(A force induced by the earths rotation). Sensors measure the degree of deformation, which correlates directly with the mass flow rate of the fluid, providing highly accurate flow rate measurements regardless of fluid properties.

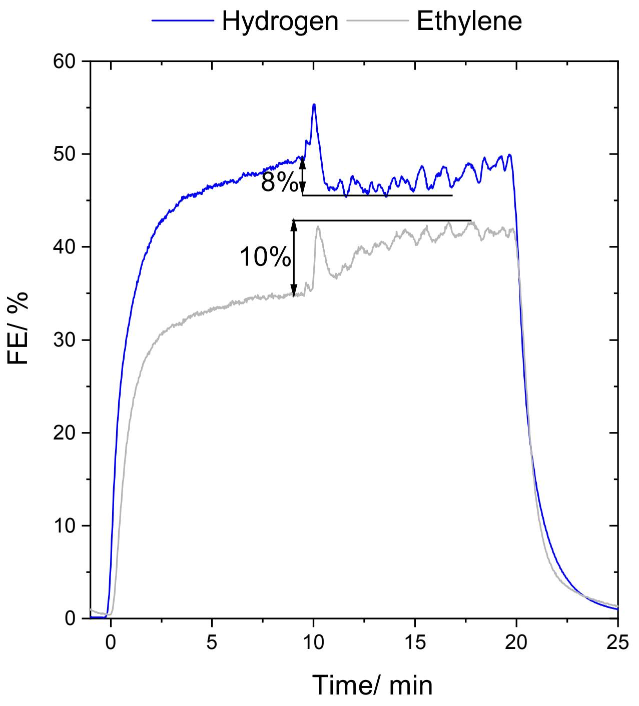

Figure S3. The change of Faradaic efficiencies in ethylene and hydrogen formation after injecting 1 M of concentrated diammonium with the octylene substituent in 1 M $KHCO_3$. Injection time: 10 min.

Considering the stabilized values, the FE increased by approximately 10 % for ethylene, and the hydrogen evolution reaction was reduced by 3 % after the injection. However, it is known that the selectivity towards H2 is steadily increasing during regular CO_2 electrolysis due to carbonation and GDE flooding. Had the partial current density of hydrogen been more stable, the real effect would have been more evident.

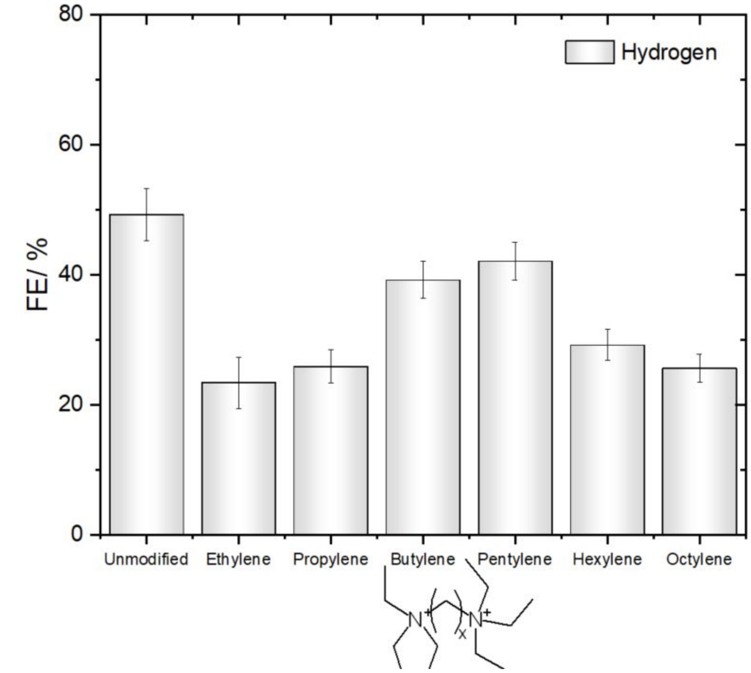

Figure S4. FE for HER during the one-hour-long CO_2RR experiments. Electrode is 1.3 mg/cm^{-2} of Cu nanoparticles (20-30 nm).Electrolyte: 1 M $KHCO_3$. Additive: 10 mM diammonium salts Current density: −400 mA cm^{-2}.

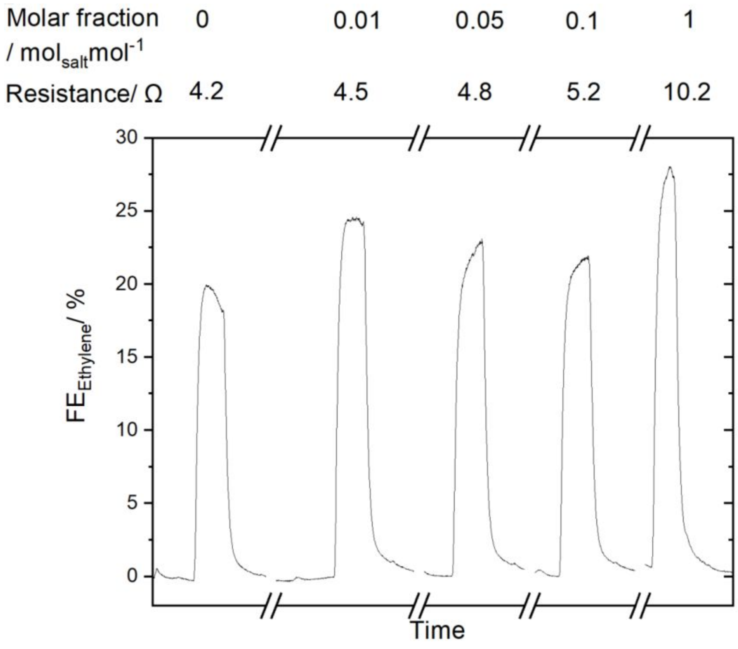

Figure S5. Ethylene FE employing 10mM of the octylene diammonium salt at different molar fractions, keeping the ionic strength at 0.5 by adding $KHCO_3$. The experiments were done consecutively, reusing the same electrode in a row and applying 30 s current steps. Cell resistance and additive molar fraction values are displayed at the top to specify the conditions in every experiment step. The values of cell resistance were estimated from EIS before every current step. Electrode: 1.3 mg/cm^{-2} of Cu nanoparticles (20-30 nm).Electrolyte: 1 M $KHCO_3$. Additive: 10 mM Current density: -400 mA cm^{-2}.

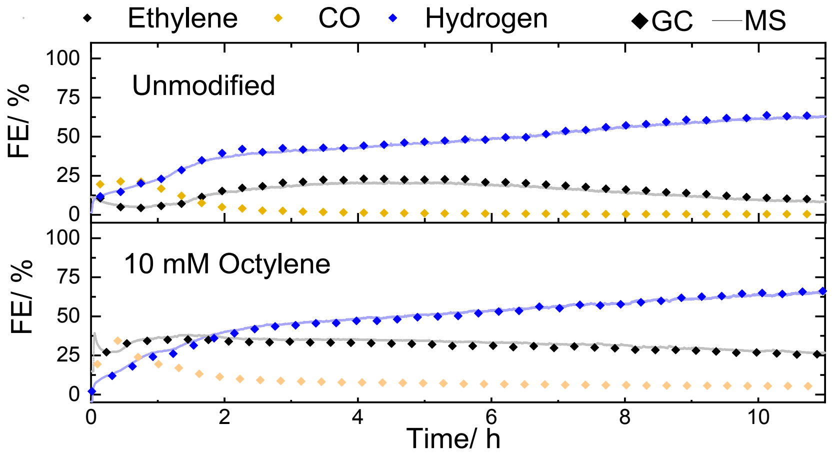

Figure S6. FE of CO_2RR gaseous products was measured using an operando GC (scatter) and real-time analysis with a mass spectrometer (line) for 10 h. Electrode: 1.3 mg cm^{-2} of Cu nanoparticles (20-30 nm).Electrolyte: 1 M $KHCO_3$. Additive: 10 mM Current density: −400 mA cm^{-2}.

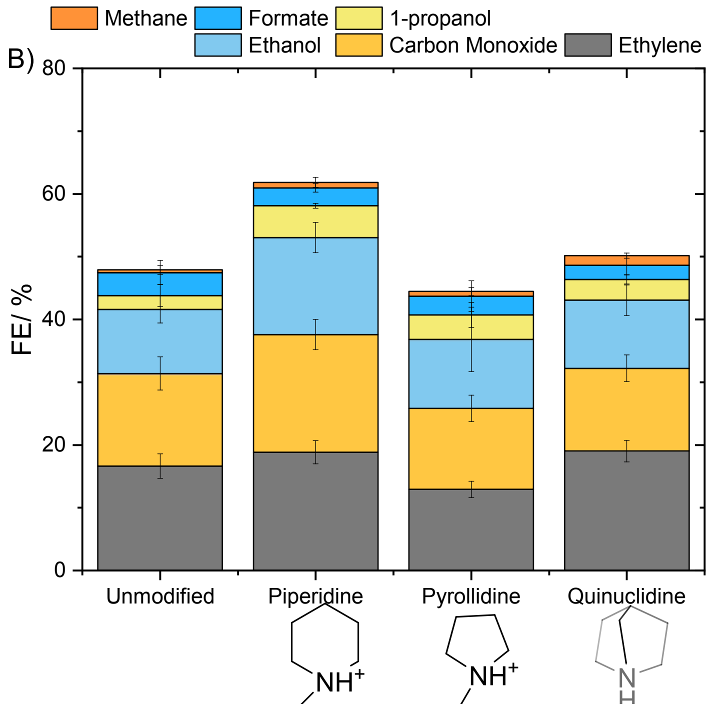

Figure S7. FE of the main CO_2RR products using alternative diammonium salts connected with an octylene chain. The chemical formulas of the different compounds are displayed next to their names for a better understanding. Electrolyte: 1 M $KHCO_3$. Additive: 10 mM Current density: −400 mA cm^{-2}.

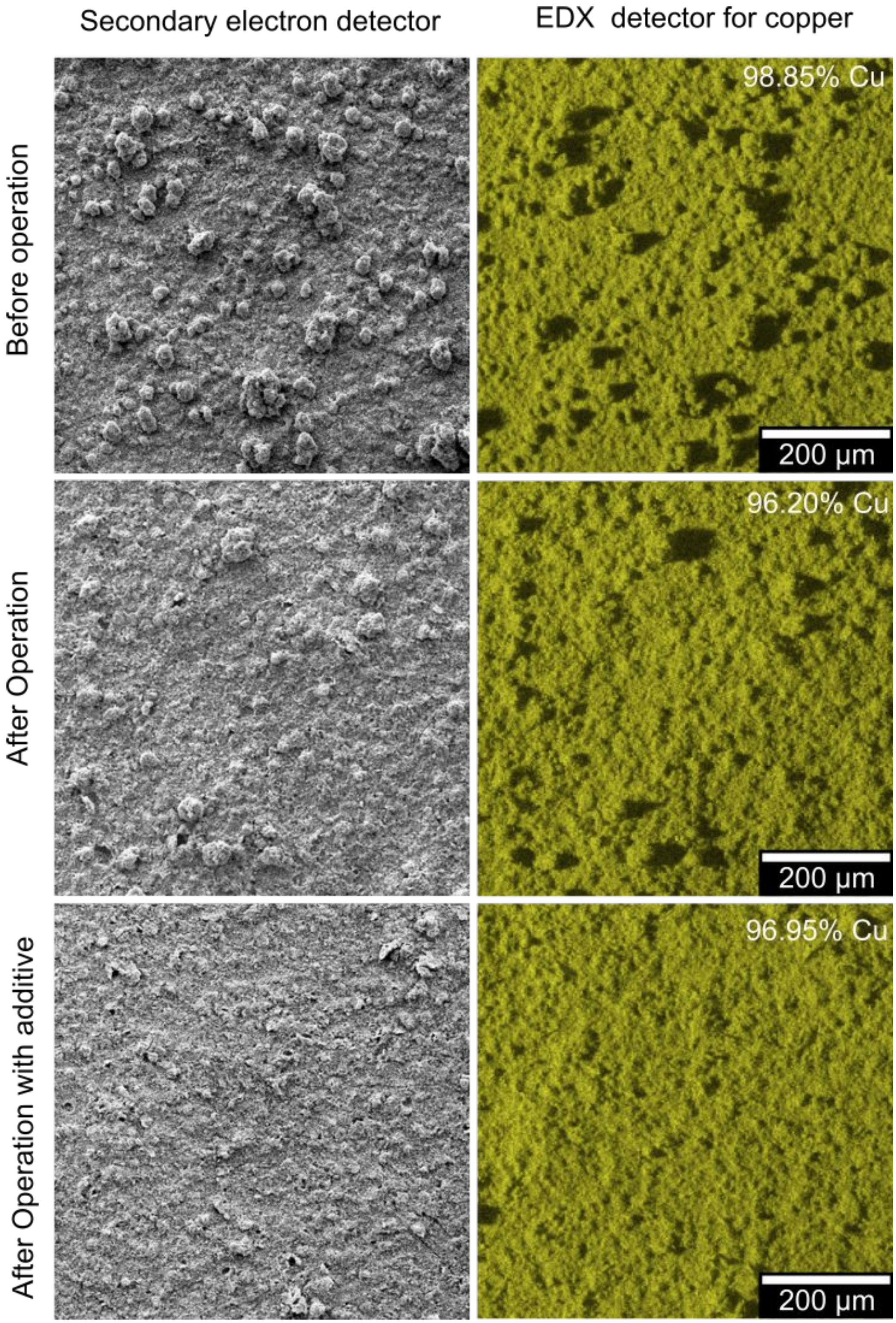

Figure S8. Scanning Electrode Microscopy (SEM) and Energy Dispersive X-Ray (EDX) images of unused catalysts (before operation) to the ones used for an hour-long operation at 400 mA cm^{-2} in 1M $KHCO_3$ (After operation). The diammonium cation used as as additive was the one with an octylene linker.

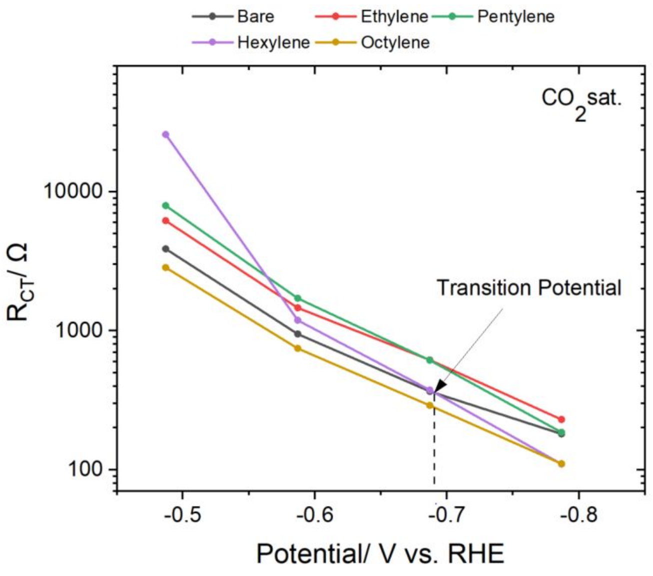

Figure S9. Fitted Charge transfer resistances at a range of potentials for CO_2RR using diammonium modifiers. Electrolyte: 1 M $KHCO_3$. Additive: 10 mM Cell: H-Cell Electrode: polycrystalline copper.

REFERENCES

(1) Hori, Y.; Wakebe, H.; Tsukamoto, T.; Koga, O. Adsorption of CO accompanied with simultaneous charge transfer on copper single crystal electrodes related with electrochemical reduction of CO2 to hydrocarbons. Surf. Sci. 1995, 335, 258-263.

(2) Zhou, Y.; Che, F.; Liu, M.; Zou, C.; Liang, Z.; De Luna, P.; Yuan, H.; Li, J.; Wang, Z.; Xie, H. Dopant-induced electron localization drives CO2 reduction to C2 hydrocarbons. Nature chemistry 2018, 10 (9), 974-980.

(3) Loiudice, A.; Lobaccaro, P.; Kamali, E. A.; Thao, T.; Huang, B. H.; Ager, J. W.; Buonsanti, R. Tailoring copper nanocrystals towards C2 products in electrochemical CO2 reduction. Angew. Chem. Int. Ed. 2016, 55 (19), 5789-5792.

(4) Ma, W.; Xie, S.; Liu, T.; Fan, Q.; Ye, J.; Sun, F.; Jiang, Z.; Zhang, Q.; Cheng, J.; Wang, Y. Electrocatalytic reduction of CO2 to ethylene and ethanol through hydrogen-assisted C–C coupling over fluorine-modified copper. Nat. Catal 2020, 3 (6), 478-487.

(5) Gao, D.; Zegkinoglou, I.; Divins, N. J.; Scholten, F.; Sinev, I.; Grosse, P.; Roldan Cuenya, B. Plasma-activated copper nanocube catalysts for efficient carbon dioxide electroreduction to hydrocarbons and alcohols. ACS nano 2017, 11 (5), 4825-4831.

(6) Mi, Y.; Shen, S.; Peng, X.; Bao, H.; Liu, X.; Luo, J. Selective electroreduction of CO2 to C2 products over Cu3N-derived Cu nanowires. ChemElectroChem 2019, 6 (9), 2393-2397.

(7) García de Arquer, F. P.; Dinh, C.-T.; Ozden, A.; Wicks, J.; McCallum, C.; Kirmani, A. R.; Nam, D.-H.; Gabardo, C.; Seifitokaldani, A.; Wang, X. CO2 electrolysis to multicarbon products at activities greater than 1 A cm– 2. Science 2020, 367 (6478), 661-666.

(8) Zhong, Y.; Xu, Y.; Ma, J.; Wang, C.; Sheng, S.; Cheng, C.; Li, M.; Han, L.; Zhou, L.; Cai, Z. An artificial electrode/electrolyte interface for CO2 electroreduction by cation surfactant self-assembly. Angew. Chem. 2020, 132 (43), 19257-19263.

(9) Tao, Z.; Wu, Z.; Wu, Y.; Wang, H. Activating copper for electrocatalytic CO2 reduction to formate via molecular interactions. ACS Catal. 2020, 10 (16), 9271-9275.

(10) Banerjee, S.; Zhang, Z.-Q.; Hall, A. S.; Thoi, V. S. Surfactant perturbation of cation interactions at the electrode–electrolyte interface in carbon dioxide reduction. ACS Catal. 2020, 10 (17), 9907-9914.
(11) Banerjee, S.; Han, X.; Thoi, V. S. Modulating the electrode–electrolyte interface with cationic surfactants in carbon dioxide reduction. ACS Catal. 2019, 9 (6), 5631-5637.
